# Psychological distress and its effect on tuberculosis treatment outcomes in Ethiopia

**DOI:** 10.3402/gha.v8.29019

**Published:** 2015-11-24

**Authors:** Habteyes Hailu Tola, Davoud Shojaeizadeh, Gholamreza Garmaroudi, Azar Tol, Mir Saeed Yekaninejad, Luche Tadesse Ejeta, Abebaw Kebede, Mehrdad Karimi, Desta Kassa

**Affiliations:** 1 Department of Health Education and Promotion, School of Public Health, Tehran University of Medical Sciences International Campus, Tehran, Iran; 2 TB/HIV Directorate, Ethiopian Public Health Institute, Addis Ababa, Ethiopia; 3 Department of Epidemiology and Biostatics, School of Public Health, Tehran University of Medical Sciences International Campus, Tehran, Iran

**Keywords:** psychological distress, treatment outcome, psychological distress trend, tuberculosis

## Abstract

**Background:**

Psychological distress is the major comorbidity among tuberculosis (TB) patients. However, its magnitude, associated factors, and effect on treatment outcome have not been adequately studied in low-income countries.

**Objective:**

This study aimed to determine the magnitude of psychological distress and its effect on treatment outcome among TB patients on treatment.

**Design:**

A follow-up study was conducted in Addis Ababa, Ethiopia, from May to December 2014. Patients (*N=*330) diagnosed with all types of TB who had been on treatment for 1–2 months were enrolled consecutively from 15 randomly selected health centers and one TB specialized hospital. Data on sociodemographic variables and economic status were collected using a structured questionnaire. The presence of psychological distress was assessed at baseline (within 1–2 months after treatment initiation) and end point (6 months after treatment initiation) using the 10-item Kessler (K-10) scale. Alcohol use and tobacco smoking history were assessed using WHO Alcohol Use Disorder Identification Test and Australian Smoking Assessment Checklist, respectively. The current WHO TB treatment outcome definition was used to differentiate the end result of each patient at completion of the treatment.

**Results:**

The overall psychological distress was 67.6% at 1–2 months and 48.5% at 6 months after treatment initiation. Multiple logistic regression analysis revealed that past TB treatment history [adjusted odds ratio (AOR): 3.76; 95% confidence interval (CI): 1.67–8.45], being on anti-TB and anti-HIV treatments (AOR: 5.35; 95% CI: 1.83–15.65), being unmarried (AOR: 4.29; 95% CI: 2.45–7.53), having alcohol use disorder (AOR: 2.95; 95% CI: 1.25–6.99), and having low economic status (AOR: 4.41; 95% CI: 2.44–7.97) were significantly associated with psychological distress at baseline. However, at 6 months after treatment initiation, only being a multidrug-resistant tuberculosis (MDR-TB) patient (AOR: 3.02; 95% CI: 1.17–7.75) and having low economic status (AOR: 3.75; 95% CI: 2.08–6.74) were able to predict psychological distress significantly. Past TB treatment history (AOR: 2.13; 95% CI: 1.10–4.12), employment status (AOR: 2.06; 95% CI: 1.06–7.00), and existence of psychological distress symptoms at 6 months after treatment initiation (AOR: 2.87; 95% CI: 1.05–7.81) were found to be associated with treatment outcome.

**Conclusions:**

The overall magnitude of psychological distress was high across the follow-up period; this was more pronounced at baseline. At baseline, past TB treatment history, being on anti-TB and anti-HIV treatments, being unmarried, and having symptoms of alcohol use disorder were associated with psychological distress. However, both at baseline and end point, low economic status was associated with psychological distress. Screening and treatment of psychological distress among TB patients across the whole treatment period is needed, and focusing more on patients who have been economically deprived, previously treated for TB, and on MDR-TB treatment are important.

Although the concept of psychological distress is still vague for some, it is broadly defined as a state of emotional suffering characterized by symptoms of depression and anxiety (1). It is a leading contributor of the total burden of disease globally (2, 3), and its comorbidity with other medical conditions is common (4, 5). Evidence shows that mortality rate due to psychological distress is high, and individuals who suffer from serious psychological distress alone die 25 years earlier than the general population (6). The combination of psychological distress with other medical conditions has several health outcomes and medicinal regimen adherence is one of those outcomes (7, 8). For instance, patients with psychological distress have a greater risk of treatment nonadherence (8–11) and are more likely to exhibit risky behaviors such as unsafe sexual practices (8, 12), tobacco smoking (13, 14), alcohol misuse (15), and suicide attempts (16) that may worsen their medical condition and even end their lives. In addition, psychological distress can affect individuals’ ability to care for their own health and can cause chronic and physical disability (17).

Existing evidence demonstrates that it is common to find comorbidity between tuberculosis (TB) and psychological distress due to their common social and medical risk factors (18, 19). The magnitude of psychological distress among TB-infected patients on treatment in developing countries is high (20–23). Studies show that depression and anxiety rates among TB patients are higher than among the general population (24). For example, in Pakistan, 72% of TB patients manifest severe or moderate forms of depression and anxiety (21). In contrast, in Nigeria 51.9% of TB patients show symptoms of psychological distress (23). Similarly, a study conducted in South Africa revealed considerable proportions of different degrees of depression among TB patients (22% with mild depression, 38% moderate, and 8% severe) (22). Still, in South Africa 81% of TB patients who were coinfected with HIV had symptoms of psychological distress (20), and 29.6% of TB–HIV coinfected patients on treatment manifested post-traumatic stress disorder (25). Moreover, a study conducted in India reported that 76% of TB patients on treatment developed common mental disorders (CMDs) (26).

Various social, medical, and individual behavioral factors are associated with psychological distress among TB patients (20–23, 26, 27). Of these, poverty or low social status, overcrowding in the home, being TB-HIV coinfected, low educational level (20), and hazardous alcohol consumption (27) are associated with psychological distress among TB patients. Moreover, TB drug-related psychological reactions (9), TB disease perception, clinical conditions (9, 20), marital status, drug adverse reaction and perceived social support (28), and gender (29) are associated with psychological distress among TB patients on treatment.

Although psychological distress has a strong effect on TB patients’ general health and treatment outcomes, few studies have been conducted to determine the magnitude and associated factors in low- and middle-income countries, including Ethiopia (20, 30). In addition, there is limited information on the effect of psychological distress on TB treatment outcome and its progress over time across treatment duration and its associated factors. Ethiopia is among 22 high TB burden countries with an estimated incidence of 258 per 100,000 population and a prevalence of 224 per 100,000 (31). However, only one study was conducted in the Oromia Region of Ethiopia to assess CMDs among TB patients coinfected with HIV, and this study reported that 64% of TB patients with HIV manifested CMDs (30). Hence, determining the magnitude of psychological distress and its associated factors among TB patients is essential to clarify the effect of TB on patients’ psychological distress. In addition, understanding the effect of psychological distress on TB treatment outcome and its progress over time is important for planning an effective intervention to improve the TB treatment success rate. Therefore, the aims of this study were as follows: 1) to assess the magnitude of psychological distress and its associated factors among TB patients on treatment; and 2) to determine the effect of psychological distress on TB treatment outcome and its trend across the treatment period in Addis Ababa, Ethiopia.

## Methods

### Study design and setting

A follow-up study was conducted among patients diagnosed with all types of TB in Addis Ababa, from May to December 2014. Currently, Addis Ababa has 53 health centers (HCs) and 10 public hospitals. The study was conducted among 15 randomly selected HCs and 1 purposively selected specialized TB hospital in Addis Ababa. The hospital was selected purposively because it is specialized in TB and a referral hospital, dedicated to treating both drug-susceptible and -resistant types of TB in the city; the hospital is easily accessible to patients with any form of TB. Addis Ababa is the capital city of Ethiopia with an estimated total population of 2,975,608 (32). All HCs in Ethiopia potentially treat TB under the directly observed treatment (DOT) strategy, and TB treatment services are free of charge (33).

### Study population and sampling

Patients with all types of TB [pulmonary, extra pulmonary, multidrug-resistant tuberculosis (MDR-TB)] being treated under the DOT strategy were the study population. Fifteen HCs were selected by simple random sampling method from ten subcities in Addis Ababa (one HC per subcity and two HCs from the two most populous subcities), and one specialized TB hospital was included purposively. Based on a previous study, a CMD magnitude of 64% (30), an estimated precision of 5%, and a 10% contingence of sample were considered for sample size estimation. As a result, 391 participants were determined to be eligible for enrollment. However, due to sample shortage during the study period, only 330 participants were enrolled consecutively, having met the criteria of being TB patients who had been on treatment for 1–2 months, not participating in any other study, being mentally capable of providing consent, being above 17 years old, and having lived in Addis Ababa for more than 6 months. The response rate of this study at both points (baseline and end point) was 100%, except for those who defaulted, transferred out, or died. Considering defaulters, deaths, and patients who transferred out as nonrespondents, the response rate of this study at end point was 90.5%.

### Data collection

A structured questionnaire was used to collect data on sociodemographic variables such as age, sex, educational status, employment status, and marital status. Economic status was assessed by one structured question with 10 options inquiring whether the participant had the following: personal house (not rented), refrigerator, cupboard, satellite dish, access to electricity and water lines, able to afford private and government house rents and electrical and water service bills, social association membership fees and able to afford the cost of eating food at least three times per day. Each option was recorded with a *yes*/*no* response; *yes* responses were given a score of 1, while *no* responses were given a score of 0. After summing up the 10 items of economic indicators of households, we divided them into low and high economic status using quartile method, which is one of the standard methods to determine the economic status of households. To collect data on psychological distress, the Kessler 10-item (K-10) scale was used (34, 35). Items on the K-10 scale measure six main psychological distress symptoms over the preceding 30 days. The frequency each item on the K-10 was experienced by a patient was recorded using a five-point Likert scale with responses ranging from ‘none of the time’ (with a lower score) to ‘all of the time’ (with a higher score). On the K-10 scale, the higher the total score, the higher the degree of psychological distress related to nonspecific depression, anxiety, and substance abuse (35). This scale has been widely used to assess CMDs among TB patients under treatment and has been validated in several settings including in Ethiopia (20, 30). Alcohol consumption history was collected using the 10-item WHO Alcohol Use Disorder Identification Test (AUDIT-10) (36), while tobacco smoking history was assessed using the West Australian Government Smoking Assessment Checklist (37). Data on HIV and antiretroviral therapy (ART) status were self-reported by patients and were cross-checked with the TB registration book. Treatment outcome, TB treatment history, and TB type were collected from the TB registration book at the end of treatment. Except for sociodemographic variables, which were measured only at baseline (within 1–2 months after treatment initiation), psychological distress and all independent variables were measured twice – both at baseline and end point (6 months after treatment initiation). Before the actual data collection, the questionnaire was validated at a selected study site by test and retest pilot study on a separate 10% of the total sample size of the main study. The consistency of test and retest measures was calculated by intraclass correlation coefficient (ICC). The ICC results for the K-10, AUDIT-10, and smoking assessment checklist were 0.93, 0.90, and 0.95, respectively. The questionnaire was administered by trained health professionals at baseline and at the end of treatment to compare the psychological distress magnitude at two points, associated factors, and its effect on treatment outcome.

### Data entry and analysis

Data was entered into IBM SPSS (Statistical Package for the Social Sciences) version 20. The magnitude of psychological distress was determined across different sociodemographic variables to show the distribution of psychological distress among participants’ characteristics. In addition, the magnitude of TB treatment outcomes and the overall psychological distress at baseline and end point were determined.

Factors associated with psychological distress were assessed by direct multiple logistic regression. Variables with a *p*-value less than 0.2 from simple logistic regression analyses were included in the final multiple logistic regression model to assess the independent effect of each variable after adjusting for potential confounders at both points. Moreover, the effect of psychological distress on TB treatment outcome was assessed with direct multiple logistic regression and its progress over the treatment period. Before running multiple logistic regression, the variance inflation factor for all predictors and the condition index for model parameters were calculated to check colinearity. For all predictors, no problem of colinearity was identified.

Although there is some doubt among different reports as to where to set the universal cutoff point for psychological distress symptoms, we used a score of 16 on the K-10 scale as the cutoff based on previous study reports (34, 35). Participants who scored 16 and above on the K-10 scale were considered to have mild to severe psychological distress, while those scoring below 16 were considered relatively asymptomatic or well. Six items of the WHO TB treatment outcome registration (*cured*, *treatment completed*, *lost to follow-up*, *treatment failed*, *died*, and *not evaluated*) were used to report the final result of each participant. In addition, *treatment success* was considered the sum of *cured* and *treatment completed*, and *lost to follow-up*, *treatment failed*, and *died* were considered poor treatment outcomes.

### Ethical consideration

Ethical approval was obtained from the research ethical review boards of the Tehran University of Medical Sciences International Campus, Ethiopian Public Health Institute, Addis Ababa City Administration Health Bureau, and St. Peter TB Specialized Hospital. Both oral and written informed consent were obtained from each study participant. Study participants with severe psychological distress were counseled within health facilities by trained health professionals or referred to other higher health facilities for further treatment.

## Results

### Study participants’ characteristics

A total of 330 TB patients on treatment were enrolled in the study. More than half [191 (57.9%)] of participants were male with a mean age of 32.21 years (SD±=12.00 years), and the age range was 18 to 90 years. The majority of participants (65.2%) were below 35 years of age, 28 (8.5%) were current smokers, 177 (53.6%) had elementary schooling or less, 211 (63.9%) were unmarried, 153 (46.4%) were unemployed, and 115 (34.8%) were working with the government or private companies, while 10.7% were self-employed. Four-fifths (81.2%) of participants had no alcohol use disorder and 243 (73.6%) were in the category of low economic status. Three-fourths (77.9%) of participants were new to TB treatment, 41 (12.4%) were HIV seroreactive, and 52 (15.8%) were on dual treatment (anti-TB and anti-HIV). More than half of the participants (57.0%) were diagnosed with pulmonary TB, while 89 (27.0%) and 53 (16.1%) were diagnosed with extrapulmonary TB and MDR-TB, respectively. Regarding treatment outcomes, 55 (16.7%) participants were cured, 182 (55.2%) completed their treatment, 16 (4.8%) defaulted from the treatment, 8 (2.4%) died, and 11 (3.3%) transferred out to other treatment sites. In addition, treatment for 12 (3.6%) participants failed and the treatment outcomes of 46 (13.9%) participants were unknown, because they were on MDR-TB treatment, which requires more than 18 months of follow-up to know the treatment outcome. The overall treatment success rate (*cured* plus *treatment completed*) was 235 (71.2%) and the overall poor treatment outcome (the sum of *treatment failed*, *lost to follow-up*, and *died*) was 36 (10.8%).

### Psychological distress and its distribution among participants’ characteristics

**Fig. 1:**
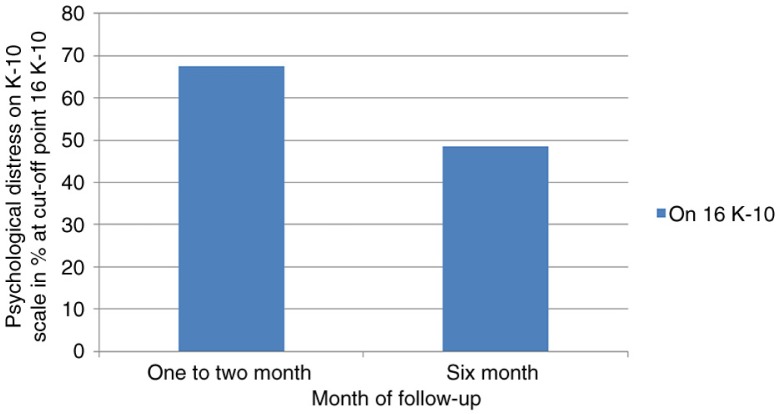
Magnitude of psychological distress at 1–2 and 6 months after treatment initiation.

Psychological distress characterized by depression and/or anxiety at enrollment (1–2 months after treatment initiation) and at 6 months after treatment initiation is displayed in Table 1. The overall prevalence of mild to severe psychological distress symptoms was 67.6% at enrollment and 48.5% at 6 months after treatment initiation (Fig. 1). At enrollment, psychological distress symptoms were prevalent among participants with the following characteristics: male gender, 129 (57.8%); age above 35 years, 74 (76.3%); current smokers, 24 (85.7%); patients who were previously treated for TB, 64 (87.7%); HIV-seroreactive patients, 37 (90.2%); those on dual-treatment (anti-TB and anti-HIV), 47 (90.4%); unmarried individuals, 163 (77.3%); those who had alcohol use disorder, 54 (87.1%); and individuals with low economic status, 186 (76.5%) (Table 1). The distribution of participants’ characteristics of psychological distress was approximately similar at baseline and 6 months after treatment initiation (Table 1). Participants who had been on treatment for 1–2 months were more likely to report psychological distress symptoms than participants who had been on treatment for 6 months (*p*<0.001). Thus, the overall psychological distress magnitude decreased across the treatment period (Fig. 1).

**Table 1:** Distribution of psychological distress across participant characteristics at baseline

		Psychological distress symptoms at 2 months (*N*=330)	
		
Variable		No symptoms *N* (%)	Symptoms exist *N* (%)
Gender	Male	62 (32.5)	129 (67.5)
	Female	45 (32.4)	94 (67.6)
Age group (in years)	<35	84 (36.2)	148 (63.8)
	≥35	23 (23.7)	74 (76.3)
Smoking history	Smoker	4 (14.3)	24 (85.7)
	Nonsmoker	103 (34.1)	199 (65.9)
TB treatment history	First time treated	98 (38.1)	159 (61.9)
	Previously treated	9 (12.3)	64 (87.7)
HIV status	Nonreactive	103 (35.6)	186 (64.4)
	Reactive	4 (9.8)	37 (90.2)
Marital status	Married	59 (49.6)	60 (50.4)
	Unmarried	48 (22.7)	163 (77.3)
ART status	Not on ART	102 (36.7)	176 (63.3)
	On ART	5 (9.6)	47 (90.4)
Education status	High school or less	92 (32.6)	190 (67.4)
	Diploma or above	15 (31.2)	33 (68.8)
TB type	PTB	53 (28.2)	135 (71.8)
	MDR-TB	16 (30.2)	37 (69.8)
	EPTB	38 (42.7)	51 (57.3)
Employment status	Daily labor	11 (40.7)	16 (59.3)
	Unemployed	44 (28.8)	109 (71.2)
	Employed	52 (34.7)	98 (65.3)
Alcohol use disorder risk	No risk	99 (36.9)	169 (63.1)
	Risk exists	8 (12.9)	54 (87.1)
Economic status	Low	57 (23.5)	186 (76.5)
	High	50 (57.5)	37 (42.5)

TB, tuberculosis; ART, antiretroviral therapy; PTB, pulmonary TB; MDR-TB, multidrug-resistant tuberculosis; EPTB, extra pulmonary TB.

### Factors associated with psychological distress

The association of different factors with psychological distress was assessed with simple logistic regression analysis and displayed in odds ratio [95% confidence interval (CI)]. Participants who were above 35 years old [crude odds ratio (COR): 1.83; 95% CI: 1.07–3.13], were smokers (COR: 3.11; 95% CI: 1.05–9.19), had previously been treated for TB (COR: 4.33; 95% CI: 2.09–9.20), were on both TB and HIV treatment (COR: 5.45; 95% CI: 2.10–14.14), had alcohol use disorder (COR: 3.95; 95% CI: 1.81–8.65), and had low economic status (COR: 4.41; 95% CI: 2.63–7.41) were more likely to be psychologically distressed at both baseline (1–2 months) and 6 months after treatment initiation. Moreover, HIV-seroreactive status (COR: 5.12; 95% CI: 1.78–14.78) and marital status (COR: 3.34; 95% CI: 2.06–5.41) were significantly associated with psychological distress at baseline, but not at 6 months after treatment initiation (Table 2). In addition, being on MDR-TB treatment (COR: 2.77; 95% CI: 1.13–6.80) was not significantly associated with psychological distress at baseline, but was strongly associated 6 months after treatment initiation (Table 2). However, the remaining participant characteristics were not significantly associated with psychological distress, either at enrollment or 6 months after treatment initiation (Table 2).

**Table 2:** Predictors of psychological distress at baseline and 6 months (K-10>16)

		At enrollment (*N*=330)		At 6 months (*N*=298)	
			
Variable		Crude OR (95% CI)	Adjusted OR (95% CI)	Crude OR (95% CI)	Adjusted OR (95% CI)
Gender	Female	1.00		1.00	
	Male	1.00 (0.62–1.59)		1.44 (0.85–2.44)	1.32 (0.77–2.26)
Age group	<35 years	1.00		1.00	
	≥35 years	1.83 (1.07–3.13)*	1.35 (0.76–2.39)	2.24 (1.16–4.33)*	1.81 (0.89–3.82)
Smoking history	Nonsmoker	1.00		1.00	
	Smoker	3.11 (1.05–9.19)*	1.37 (0.39–4.86)	8.35 (1.11–62.97)*	4.04 (0.49–33.10)
TB treatment history	First time treated	1.00		1.00	
	Previously treated	4.33 (2.09–9.20)*	3.76 (1.67–8.45)*	2.18 (1.05–4.54)*	1.56 (0.73–3.36)
HIV status	Nonreactive	1.00		1.00	
	Reactive	5.12 (1.78–14.78)*	2.01 (0.71–6.92)	2.81 (0.96–8.25)	1.76 (0.34–8.99)
ART status	Not on ART	1.00		1.00	
	On ART	5.45 (2.10–14.14)*	5.35 (1.83–15.65)*	3.00 (1.14–7.91)*	1.47 (0.33–6.51)
Marital status	Married	1.00		1.00	
	Unmarried	3.34 (2.06–5.41)*	4.29 (2.45–7.53)*	1.28 (0.74–219)	
Education status	Diploma or above	1.00			
	High school or less	0.94 (0.49–1.82)		0.94 (0.44–2.01)	
TB type	Drug suspect TB	1.00		1.00	
	MDR-TB	1.13 (0.60–2.14)		2.77 (1.13–6.80)*	3.02 (1.17–7.75)*
Employment status	Employed	1.00		1.00	
	Unemployed	1.21 (0.76–1.92)		1.24 (0.73–2.10)	1.04 (0.59–1.83)
AUD symptoms	No symptoms	1.00		1.00	
	Symptoms exist	3.95 (1.81–8.65)*	2.95 (1.25–6.99)*	3.57 (1.36–9.35)*	2.17 (0.78–6.03)
Economic status	High	1.00		1.00	
	Low	4.41 (2.63–7.41)*	4.41 (2.44–7.97)*	3.88 (2.21–6.80)*	3.75 (2.08–6.74)*

* Statistically significant. K-10, the 10-item Kessler scale; OR, odds ratio; CI, confidence interval; AUD, alcohol use disorder; ART: antiretroviral therapy.

Eight variables (age, current smoking history, TB treatment history, HIV-seroreactive status, ART status, marital status, alcohol use history, and economic status) with *p*-values less than 0.2 from simple logistic regression analysis were included in the final multiple logistic regression model. The model as a whole explained between 25.5% (Cox and Snell R-squared) and 35.5% (Nagelkerke R-squared) of the variance in psychological distress symptoms, and classified 76.1% of cases correctly. On final multiple logistic regression analysis, previous TB treatment history [adjusted odds ratio (AOR): 3.76; 95% CI: 1.67–8.45], being on anti-TB and anti-HIV treatments (AOR: 5.35; 95% CI: 1.83–15.65), being unmarried (AOR: 4.29; 95% CI: 2.45–7.53), having alcohol use disorder (AOR: 2.95; 95% CI: 1.25–6.99), and having low economic status (AOR: 4.41; 95% CI: 2.44–7.97) were significantly associated with psychological distress at enrollment. However, current smoking history (AOR: 1.10; 95% CI: 0.29–4.10) did not persist in predicting psychological distress significantly on multivariate analysis at enrollment.

In contrast, the model containing eight variables (age, current smoking, TB treatment history, HIV serostatus, ART status, TB type, alcohol use disorder, and economic status) with *p*-values less than 0.2 from simple logistic regression analysis at 6 months after treatment initiation explained between 12.9% (Cox and Snell R-squared) and 19.1% (Nagelkerke R-squared) of the variance in psychological distress symptoms and classified 75.4% of cases correctly. After adjusting for potential confounders, only being on MDR-TB treatment (AOR: 3.02; 95% CI: 1.17–7.75) and having low economic status (AOR: 3.75; 95% CI: 2.08–6.74) were able to predict psychological distress significantly at 6 months after treatment initiation; none of the other variables significantly predicted psychological distress at 6 months (Table 2).

### The effect of psychological distress on TB treatment outcome

#### Predictors of TB treatment outcome

Previous TB treatment history (COR: 2.15; 95% CI: 1.24–3.71), HIV-seroreactive status (COR: 2.00; 95% CI: 1.01–3.90), employment status (COR: 2.30; 95% CI: 1.39–3.82), alcohol use disorder (COR: 1.82; 95% CI: 1.02–3.25), and existence of mild to severe psychological distress symptoms at 6 months after treatment initiation (COR: 4.82; 95% CI: 1.85–12.54) were able to predict TB treatment outcome significantly from simple logistic regression analysis (Table 3). The final multiple logistic regression model containing six variables (TB treatment history, HIV serostatus, employment status, alcohol use history, psychological distress at 6 months after treatment initiation) after controlling for potential confounder explained between 14.3% (Cox and Snell R-squared) and 22.2% (Nagelkerke R-squared) of the variance in TB treatment outcome and classified 81.5% of cases correctly. Final multiple logistic regression analysis showed previous TB treatment history (AOR: 2.13; 95% CI: 1.10–4.12), employment status (AOR: 2.06; 95% CI: 1.06–7.00), and existence of psychological distress symptoms at 6 months after treatment initiation (AOR: 2.87; 95% CI: 1.05–7.81) were found to be significant predictors of TB treatment outcome (Table 3). However, HIV-seroreactive status (AOR: 2.10; 95% CI: 0.90–4.89) and existence of alcohol use disorder (AOR: 1.25; 95% CI: 0.59–2.65) did not continue to predict TB treatment outcome significantly on multivariate analysis model (Table 3). In addition, psychological distress symptoms at 1–2 months after treatment initiation were not a significant predictor of TB treatment outcome (Table 3).

**Table 3:** Predictors of TB treatment success

Variable		Crude OR (95% CI)	Adjusted OR (95% CI)
Gender	Female	1.00	
	Male	1.30 (0.79–2.13)	
Age group (in years)	<35	1.00	
	≥35	1.29 (0.77–2.16)	
Smoking history	Nonsmoker	1.00	
	Smoker	1.45 (0.65–3.31)	
TB treatment history	First time treated	1.00	
	Previously treated	2.15 (1.24–3.71)*	2.13 (1.10–4.12)*
HIV status	Nonreactive	1.00	
	Reactive	2.00 (1.01–3.90)*	2.10 (0.90–4.89)
Marital status	Married	1.00	
	Unmarried	1.26 (0.76–2.10)	
ART status	Not on ART	1.00	
	On ART	1.59 (0.85–2.96)	
Education status	High school or less	1.00	
	Diploma or above	1.19 (0.61–2.31)	
Employment status	Employed	1.00	
	Unemployed	2.30 (1.39–3.82)*	2.06 (1.06–7.00)*
Alcohol use disorder symptoms	No risk	1.00	
	Risk exists	1.82 (1.02–3.25)*	1.25 (0.59–2.65)
Economic status	High	1.00	
	Low	1.13 (0.65–1.95)	
Psychological distress at 1–2 months after treatment initiation	No symptoms	1.00	
	Symptoms exist	1.25 (0.74–2.10)	
Psychological distress at 6 months after treatment initiation	No symptoms	1.00	
	Symptoms exist	4.82 (1.85–12.54)*	2.87 (1.05–7.81)*

TB, tuberculosis; OR, odds ratio; CI, confidence interval; ART: antiretroviral therapy;

* significant variables.

## Discussion

The comorbidity of psychological distress with chronic diseases is common, and numerous associated factors exacerbate comorbidity among patients who have psychological distress with other medical conditions (5). In this study, the magnitude of psychological distress was quite high among TB patients on treatment; however, it decreased across the treatment period. The decline of psychological distress at the end point may be due to the effect of TB treatment, which might have made the symptoms related to TB disappear or decrease. The multiple logistic regression model revealed that past TB treatment history, being on anti-TB and anti-HIV treatment, being unmarried, having symptoms of alcohol use disorder, and being in a lower economic category were associated with psychological distress at enrollment. However, at end point, only being on MDR-TB treatment and being in a lower economic category were associated with psychological distress. At end point of TB treatment, the psychological distress and economic status of study participants were associated with TB treatment success.

### Psychological distress and its associated factors

The magnitude of psychological distress decreased significantly across the treatment period among TB patients on treatment. This finding was similar to that reported in a study in Ethiopia by Deribew et al. (38). Overall psychological distress symptoms were high, both at baseline of the study (67.6%) and at 6 months after treatment initiation (48.5%). Our findings were similar to those reported in the study from the Oromia Region of Ethiopia (30), which found that 64% of TB patients with HIV displayed symptoms of psychological distress. However, this finding differed from that in the study by similar authors in Ethiopia (38) at 6 months after treatment. In our study, the magnitude of psychological distress was 48.5% at 6 months; however, Deribew et al. (38) reported a psychological distress magnitude of 18.1% at 6 months after treatment initiation. This difference may be due to a difference in the cutoff point used to categorize the existence of psychological distress symptoms. We used 16 as the cutoff point for the K-10 scale, but in the case of Deribew et al. (38) the cutoff point was unclear. In addition, the magnitude of psychological distress symptoms in this study was lower than in the study reported from South Africa by Peltzer et al. (20), which found 81%. This difference is most probably due to the difference in study population, socioeconomic status, and timing of the interviews. In our study, the baseline interview was administered within 1–2 months of treatment initiation; however in the case of Peltzer et al. (20) it was administered within 1 month of TB treatment initiation. At 1 month of treatment, the symptoms of psychological distress may be high, because patients recently diagnosed with TB might have developed anxiety immediately upon learning their diagnosis, and symptoms related to TB disease are less likely to decrease within 1 month of treatment initiation.

Although we were not able to find a sufficiently similar study to compare to our findings on the association between previous TB treatment history and psychological distress symptoms, one study reported by Peltzer and Louw (39) showed an association between previous TB treatment history and suicide ideation. This finding is consistent with our finding in showing that individuals who had previously been treated for TB were 3.76 times more likely to report psychological distress than those who were newly diagnosed and had just begun TB treatment.

According to a systematic review study conducted by Pachi et al. (9), TB drugs themselves can induce a psychological reaction and put patients under psychological distress. In addition, antiretroviral drugs, particularly efavirenz, can induce psychogenic effects (19). These findings were similar to our own finding that being on dual treatment (anti-TB and anti-HIV) was associated with psychological distress within 1–2 months of treatment initiation. On the contrary, the study conducted in the Oromia Region of Ethiopia showed the absence of association between being on ART and CMDs (30).

Masumoto et al. (28) found cohabitation status to be associated with psychological distress. This finding is similar to our finding that not being in a marital union (cohabitating, never married, divorced, or separated) was associated with psychological distress. Contrary to our finding, Deribew et al. (30) reported that marital status was not associated with CMDs. This difference may be due to the difference in study participants in both studies. All participants of this study were from the capital city where the living cost is generally too high compared with other urban areas in Ethiopia, which could contribute to higher psychological distress. However, in the case of Deribew et al. (30) the study population was from a semi-urban area where socioeconomic problems are relatively fewer.

A multicenter study carried out by Theron et al. (40) showed a strong association between an increased psychological distress score on the K-10 and heavy alcohol use. In addition, the study conducted by Deribew et al. (30) in the Oromia Region of Ethiopia revealed that the use of a locally made alcoholic beverage (*katikala*) by economically disadvantaged TB patients was associated with CMDs. These findings were in agreement with ours in that participants who had an alcohol use disorder according to the WHO AUDIT-10 were 3.57 times more likely to show psychological distress symptoms. The occurrence of psychological distress symptoms among TB patients who had alcohol use disorder based on the WHO AUDIT-10 criteria may be due to alcohol use disorder or other socioeconomic factors.

Individuals in developing countries suffer from the influence of economic stress, which leads to psychological distress (41). Poverty is one of the main factors associated with psychological distress among TB patients in low- and middle-income countries (20, 30). In this study, the economic status of TB patients was strongly associated with psychological distress, both at baseline and end point of the treatment. In addition, 6 months after treatment initiation, participants on MDR-TB treatment were more likely to report psychological distress than participants who were on first-line TB treatment. However, at baseline of treatment initiation, there was no significant difference observed between patients on MDR-TB treatment and those on first-line TB treatment with respect to psychological distress. This difference may be due to the fact that as the time on treatment increases, psychological reactions to MDR-TB drugs increase; or it may be that patients become frustrated with the longer period required for MDR-TB treatment. Although we were unable to find previous similar studies, a study carried out by Pachi et al. (9) reported that TB medication itself had an effect of ‘psychological manifestation’.

### The effect of psychological distress on TB treatment outcome

In this study, treatment success rate (*cured* 16.1% plus *treatment completed* 55.2%) was 71.3%. This result was consistent with a retrospective study conducted in southern Ethiopia by Cuevas et al. (42), who reported 74.8% treatment success. On the contrary, studies conducted in northern Ethiopia by Berhe et al. (43) and in southern Ethiopia by Datiko and Lindtjørn (44) showed higher treatment success rates, reporting 89.2 and 83.1%, respectively. Similarly, a 5-year retrospective study conducted by Endris et al. (45) in northern Ethiopia demonstrated a higher (94.8%) treatment success rate than ours. In addition, an historical cohort study conducted in Addis Ababa reported an 82.7% (18.1% *cured* plus 64.6% *treatment completed*) treatment success rate (46), which is still higher than our finding. This difference in treatment success rate is most probably due to difference in study design and study population. The four studies that reported higher treatment success used a registration-based retrospective study design; however, our study was a 6-month follow-up of patients until treatment completion. In addition, unlike the study reported from Addis Ababa, the other studies were conducted in rural and semi-urban populations. It is obvious that using secondary data is prone to registration error, which leads to either overestimation or underestimation of treatment success rate. Moreover, these differences may be due to the treatment outcomes of 46 (13.9%) participants, which were unknown in our study, because these participants were on MDR-TB treatment. MDR-TB treatment requires more than 18 months of follow-up to categorize treatment outcome, but our study follow-up period was only 6 months.

In this study, previous TB treatment history, employment status, and existence of psychological distress symptoms at 6 months after treatment initiation predicted TB treatment outcome. A study in northern Ethiopia by Berhe et al. (43) found similar results to our finding that unemployment and previous TB treatment history were associated with TB treatment outcome. Although we were unable to find a previous study assessing the direct effect of psychological distress on TB treatment outcome, available studies (8–11) reported that psychological distress had an effect of TB treatment nonadherence, which in turn directly affected TB treatment outcome. These results are similar to our finding that psychological distress at 6 months after treatment initiation was associated with TB treatment outcome. In addition, according to a study by Prince et al. (19) and a systematic review by Pachi et al. (9), the association between psychological distress and other comorbidities could contribute to nonadherence to the recommended TB treatment regimens, leading to poor treatment outcomes. Treatment nonadherence is the most challenging factor for global TB control programs, as it is a direct cause of poor treatment outcomes. In addition, treatment nonadherence, which has a strong association with psychological distress, is the most disastrous phenomena: it causes treatment failure, prolongs the disease transmission period, increases the risk of needing retreatment and developing drug resistance, and in general leads to poor health quality of patients. Although the sample size of this study is slightly small, the rates of default, death, and patients transferring out to other health facilities were only 4.8%, 2.4%, and 3.6%, respectively. These figures are quite low; as a result we do not think that bias was introduced and our findings were less likely to be influenced by these attrition rates.

### Limitations of the study

The main limitation of this study was that TB patients on MDR-TB treatment were included. As a result, this situation may underestimate the treatment success rate at the study site, because the long follow-up needed for MDR-TB treatment prevented us from knowing the treatment outcomes of these patients (in our study the follow-up period was only 6 months). In addition, treatment outcomes for participants who transferred out to another treatment center were not captured, which may have made the treatment success rate at the study area seem lower than it was. Moreover, this study did not include healthy participants as a control group to distinguish psychological distress related directly to TB disease rather than other socioeconomic conditions. Hence, our findings might have underestimated the treatment success rate and might not have captured the independent magnitude of psychological distress due to TB disease only. Future similar studies need to also include a qualitative study (in addition to a quantitative study) so as to explore the precise associated factors of psychological distress among TB patients, which our study did not address.

## Conclusions

Although the overall magnitude of psychological distress among TB patients on treatment was high both at baseline and end point, the magnitude was relatively lower at end point in comparison to that at baseline. Psychological distress symptoms at 6 months after treatment initiation predicted TB treatment outcome significantly. Thus, TB treatment strategies should consider screening and treating psychologically distressed individuals among TB patients by targeting patients who have previously been treated for TB, are on anti-TB or anti-HIV treatment, are unmarried or divorced, have low economic status, are at risk for alcohol use disorder, and are on MDR-TB treatment. In addition, a rigorous comparative longitudinal study with a large sample size is needed to clearly identify the independent effect of TB disease on the mental health status of TB patients and the effect of psychological distress on treatment outcomes.
